# Arginase II Expressed in Cancer-Associated Fibroblasts Indicates Tissue Hypoxia and Predicts Poor Outcome in Patients with Pancreatic Cancer

**DOI:** 10.1371/journal.pone.0055146

**Published:** 2013-02-12

**Authors:** Yoshinori Ino, Rie Yamazaki-Itoh, Seiji Oguro, Kazuaki Shimada, Tomoo Kosuge, Jan Zavada, Yae Kanai, Nobuyoshi Hiraoka

**Affiliations:** 1 Division of Molecular Pathology, National Cancer Center Research Institute, Tokyo, Japan; 2 Hepatobiliary and Pancreatic Surgery Division, National Cancer Center Hospital, Tokyo, Japan; 3 Institute of Organic Chemistry and Biochemistry, Prague, Czech Republic; Technische Universität München, Germany

## Abstract

An adequate level of arginine in the tissue microenvironment is essential for T cell activity and survival. Arginine levels are regulated by the arginine-catabolizing enzyme, arginase (ARG). It has been reported that arginase II (ARG2), one of two ARGs, is aberrantly expressed in prostate cancer cells, which convert arginine into ornithine, resulting in a lack of arginine that weakens tumor-infiltrating lymphocytes and renders them dysfunctional. However, immune suppression mediated by ARG2-expressing cancer cells in lung cancer has not been observed. Here we studied the expression of ARG2 in pancreatic ductal carcinoma (PDC) tissue clinicopathologically by examining over 200 cases of PDC. In contrast to prostate cancer, ARG2 expression was rarely demonstrated in PDC cells by immunohistochemistry, and instead ARG2 was characteristically expressed in α-smooth muscle actin-positive cancer-associated fibroblasts (CAFs), especially those located within and around necrotic areas in PDC. The presence of ARG2-expressing CAFs was closely correlated with shorter overall survival (OS; *P*  = 0.003) and disease-free survival (DFS; *P*  = 0.0006). Multivariate Cox regression analysis showed that the presence of ARG2-expressing CAFs in PDC tissue was an independent predictor of poorer OS (hazard ratio [HR]  = 1.582, *P*  = 0.007) and DFS (HR  = 1.715, *P*  = 0.001) in PDC patients. In addition to the characteristic distribution of ARG2-expressing CAFs, such CAFs co-expressed carbonic anhydrase IX, SLC2A1, or HIF-1α, markers of hypoxia, in PDC tissue. Furthermore, *in vitro* experiments revealed that cultured fibroblasts extracted from PDC tissue expressed the ARG2 transcript after exposure to hypoxia, which had arginase activity. These results indicate that cancer cell-mediated immune suppression through ARG2 expression is not a general event and that the presence of ARG2-expressing CAFs is an indicator of poor prognosis, as well as hypoxia, in PDC tissue.

## Introduction

The tumor microenvironment plays important roles in the biological behavior of any tumor, which includes the host immune response, tissue oxygen tension, and cancer-associated fibroblasts (CAFs) [1].

Adequate levels of arginine in the extracellular milieu are crucial for T cell proliferation and activity. [2], [3] Arginine is one of the semi-essential amino acids and arginine levels are regulated by arginase (ARG), [4] which hydrolyzes arginine to ornithine and urea. There are two isozymes of ARG, ARG1 and ARG2. ARG1, a cytoplasmic enzyme, is expressed mainly in the liver and detoxifies ammonia. ARG2 is expressed as a mitochondrial protein in a variety of tissues, such as kidney, prostate, and small intestine. Arginine also serves as a substrate for nitric oxide synthase (NOS), yielding nitric oxide (NO) and other reactive nitrogen intermediates.

It has been reported that ARG2 is aberrantly expressed in prostate cancer cells, being involved in tumor immune escape mediated by arginine consumption, resulting in a lack of arginine that weakens tumor-infiltrating lymphocytes and renders them dysfunctional. [5] Prostate cancer concomitantly expresses NOS2, thereby reducing arginine progressively and forming peroxynitrite that triggers T cell apoptosis by inhibiting the signal transduction necessary for cellular activation. [5] However, these immunosuppressive effects through the ARG2 expression in cancer cells were not evident in the next lung cancer study. More than 80% (99/120 cases) of lung cancers expressed ARG2 to variable degrees, although the expression of ARG2 had no effect on clinicopathological characteristics, including the host tumor immune response. [6] Now it is controversial, the impact of ARG2 on the clinical features of human cancers.

Pancreatic cancer [pancreatic ductal carcinoma (PDC)] is the fourth and fifth leading cause of cancer-related death in the United States and Japan, respectively. [7], [8] The overall 5-year survival rate for patients with pancreatic cancer is 3–5%, [7], [9], [10], [11] in view of its aggressive growth and early metastatic dissemination. The rate of mortality due to this cancer has shown no obvious improvement for decades. The development of predictive biomarkers to assist selection of patient subsets is useful for studies aimed at reducing the mortality of PDC patients, especially in phase clinical studies designed to evaluate various therapeutic approaches [12].

In the present study, we investigated the expression and clinicopathological significance of ARG2 in PDC. We found that only a few PDC cells expressed ARG2, but noticed that ARG2 was expressed in certain stromal cells present in PDC tissue. We also found that the presence of ARG2-expressing stromal cells in PDC tissue was a clinicopathologically significant variable, being associated with a poorer outcome, as well as an indicator of hypoxia.

## Results

### ARG2 Expression is Rare in PDC Cells, but Characteristically Expressed in Spindle-shaped Stromal Cells within and Around Necrotic Areas in PDC Tissue

**Figure 1:**
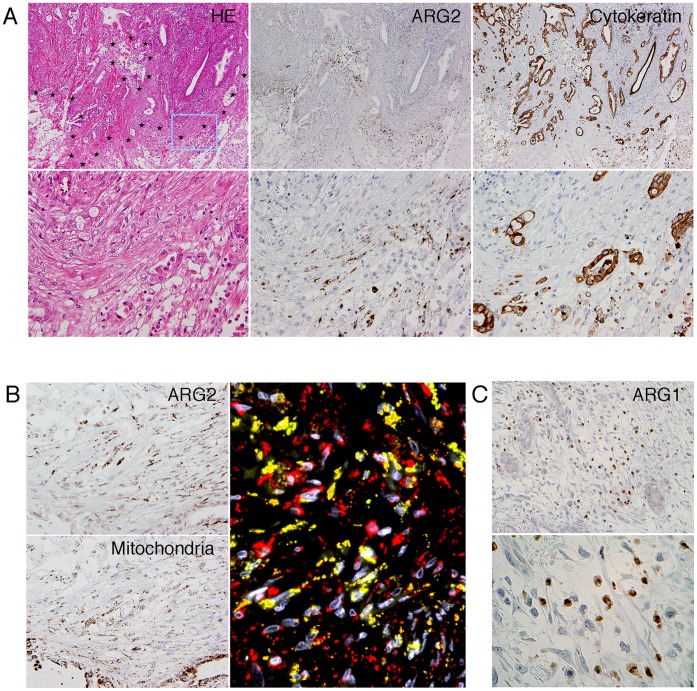
Immunohistochemical expression of ARG2 in stromal cells within and around necrotic areas in PDC tissue. (A) Histology of PDC tissue in low- (upper columns) and high-power view (lower columns). HE staining (left columns) and immunohistochemistry for ARG2 (center columns) and cytokeratins (right columns) in serial tissue sections. Necrotic areas are surrounded by star marks (large area of necrosis is located at bottom right) in the upper HE photo and the rectangle (light blue) corresponds to the area of the lower column. (B) Immunohistochemistry for ARG2 (left upper column) and for mitochondria (left lower column) in high-power views. Positive staining of ARG2 is visualized as a dot-like or coarse granular pattern in the cytoplasm of spindle cells. This positive staining pattern was compatible with mitochondrial antigen. Double immunofluorescence (right column) shows ARG2 (green), mitochondria (red), and nuclei (white). Almost all ARG2-positive staining is co-localized with mitochondria (yellow). (C) Immunohistochemical expression of ARG1 was observed only in neutrophils. Upper and lower columns are middle- and high-power views, respectively.

Immunohistochemical analysis revealed that ARG2 expression was present in a small number of PDC cases, where it was expressed focally in PDC cells. In contrast, ARG2 was expressed in spindle-shaped stromal cells within and around necrotic areas (Figure 1A). ARG2 was stained with a dot-like or coarse granular pattern in the cytoplasm of the spindle cells, compatible with the fact that ARG2 is localized in mitochondria (Figure 1B). ARG2 was also expressed in Langerhans islet cells and ganglion cells in pancreas tissue under non-pathological conditions. Expression of ARG1 was detected only in the cytoplasm of neutrophils by immunohistochemistry, and not in cancer cells (Figure 1C).

### Prognostic Significance of ARG2 Expression in Stromal Cells

**Figure 2:**
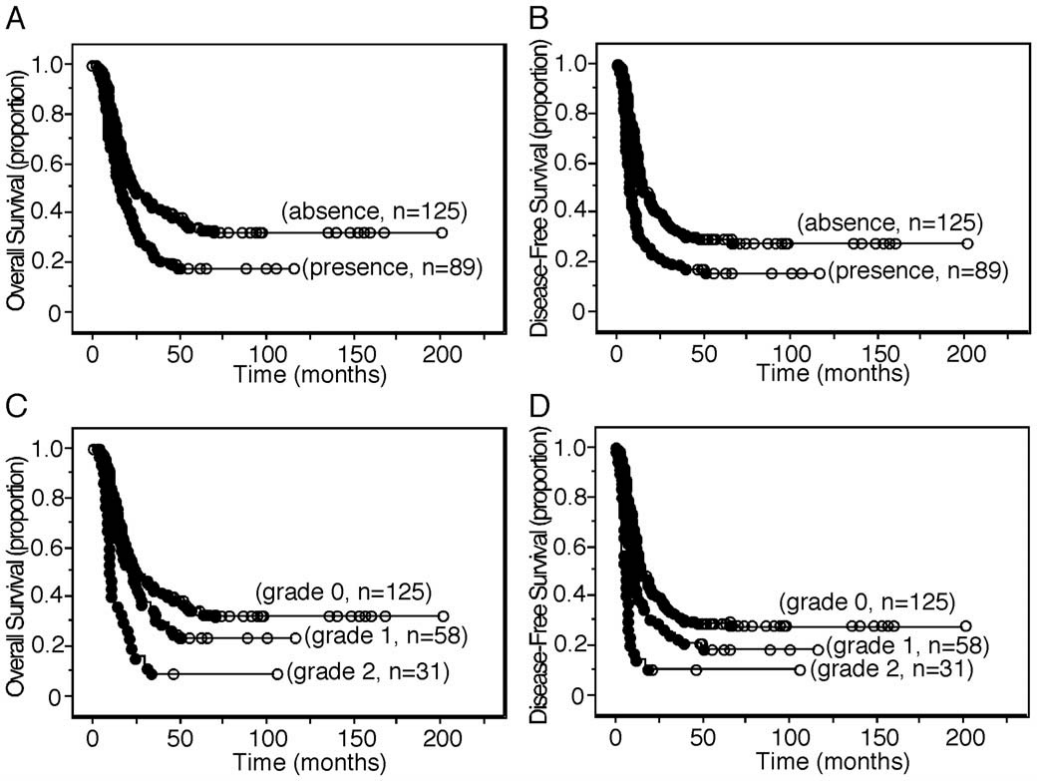
Kaplan-Meier survival curves. (A, C) Kaplan-Meier survival curve showing a comparison of overall survival between the presence (grades 1 and 2) and absence (grade 0) of ARG2 expression in stromal cells (*P*  = 0.003) in A and that among expression grades (grades 0 to 2) of ARG2 in stromal cells (grade 0 vs. grade 1, log-rank test, *P*  = 2.08; grade 0 vs. grade 2, *P*<0.0001; grade 1 vs. grade 2, *P*  = 0.0009) in C. (B, D) Kaplan-Meier survival curve showing a comparison of disease-free survival between the presence (grades 1 and 2) and absence (grade 0) of ARG2 expression in stromal cells (*P*  = 0.0006) in B and that among expression grades (grades 0 to 2) of ARG2 in stromal cells (grade 0 vs. grade 1, *P*  = 0.069; grade 0 vs. grade 2, *P*<0.0001; grade 1 vs. grade 2, *P*  = 0.013) in D. Black circle and white circle represent censoring and failure, respectively.

Survival analysis demonstrated an association between the presence of ARG2-expressing stromal cells and shorter OS (*P*  = 0.003) and DFS (*P*  = 0.0006) (Figure 2), although no association was found between the presence of ARG2-expressing cancer cells and any patient survival parameter. When the mean number of positive cells was zero, 1 to 80, and more than 80, the staining grades assigned were zero (absence), one (lower expression), and 2 (higher expression), respectively. As ARG2 expression increased, survival became significantly shorter in terms of both OS and DFS (Figure 2).

The average survivals of patients having PDC with or without ARG2-expressing stromal cells were 22.71±1.77 months and 37.13±2.41 months, respectively. One-year survival rates for patients having PDC with or without ARG2-expressing stromal cells were 66.9±5.2% and 78.7±3.7%, respectively; the 2-year survival rates were 36.1±5.4% and 51.6±4.6%, and the 5-year survival rates 17.1±4.4% and 34.5±4.5%, respectively.

Multivariate Cox regression analysis including tumor size, node status, metastasis status, tumor histological grade, margin status, nerve plexus invasion, lymphatic invasion, venous invasion, intrapancreatic neural invasion, and expression of ARG2 in stromal cells showed that expression of ARG2 in stromal cells, metastatic status, lymphatic invasion, and venous invasion were independent predictors of OS (Table 1), and that expression of ARG2 in stromal cells, metastatic status, nerve plexus invasion, and venous invasion were independent predictors of DFS (Table 2). When the expression of ARG2 in stromal cells was categorized into no and lower expression versus higher expression (see Materials and methods), univariate analysis of OS and DFS showed (*P*<0.0001; hazard ratio [HR]  = 2.505; 95% confidence interval [CI], 1.649 to 3.804) and (*P*<0.0001; HR  = 2.519; 95% CI, 1.658 to 3.827), respectively. Multivariate analysis of OS and DFS showed (*P*<0.0001; HR  = 2.687; 95% CI, 1.759 to 4.103) and (*P*<0.0001; HR  = 2.451; 95% CI, 1.608 to 3.736), respectively.

**Table 1:** Univariate and multivariate analyses of prognostic factors associated with overall survival in patients with ductal carcinoma of the pancreas.

	Univariate analysis		Multivariate analysis	
Variables	HR (95% CI)	*P* value	HR (95% CI)	*P* value
Age (≥60 years/<60 years)	0.851 (0.604–1.198)	0.355		
Gender (male/female)	1.159 (0.824–1.629)	0.397		
Tumor size (≥30 mm/<30 mm)	1.881 (1.242–2.851)	**0.003**		
Pathologic tumor status (T1+T2/T3)	5.227 (0.730–37.4)	0.100		
Pathologic node status (N0/N1)	2.046 (1.296–3.230)	**0.002**		
Pathologic metastasis status (M0/M1)	2.589 (1.589–4.217)	**0.0001**	2.160 (1.315–3.547)	**0.002**
Histological grade (W/D/M/D, P/D)*	1.539 (1.042–2.274)	**0.030**		
Tumor margin status (negative/positive)	1.528 (1.055–2.214)	**0.025**		
Nerve plexus invasion (absence/presence)*	1.626 (1.138–2.324)	**0.008**		
Lymphatic invasion (0, 1/2, 3)*	2.457 (1.637–3.686)	**<0.0001**	1.989 (1.302–3.037)	**0.002**
Venous invasion (0, 1/2, 3)*	1.966 (1.380–2.801)	**0.0002**	1.575 (1.091–2.275)	**0.015**
Intrapancreatic neural invasion (0, 1/2, 3)*	1.704 (1.210–2.399)	**0.002**		
Expression of ARG2 in PDC (absence/presence)	1.267 (0.833–1.928)	0.269		
Expression of ARG2 in stromal cells (absence/presence)	1.654 (1.188–2.304)	**0.003**	1.582 (1.134–2.209)	**0.007**

W/D, well differentiated tubular adenocarcinoma and papillary carcinoma; M/D, moderately differentiated.

tubular adenocarcinoma; P/D, poorly differentaited adenocarcinoma.

* Classified according to the classification of pancreatic carcinoma of Japan Pancreas Society.

**Table 2:** Univariate and multivariate analyses of prognostic factors associated with disease-free survival in patients with ductal carcinoma of the pancreas.

	Univariate analysis		Multivariate analysis	
Variables	HR (95% CI)	*P* value	HR (95% CI)	*P* value
Age (≥60 years/<60 years)	0.873 (0.627–1.217)	0.423		
Gender (male/female)	1.035 (0.749–1.429)	0.836		
Tumor size (≥30 mm/<30 mm)	1.983 (1.328–2.959)	**0.0008**		
Pathologic tumor status (T1+T2/T3)	4.874 (0.681–34.9)	0.115		
Pathologic node status (N0/N1)	2.078 (1.331–3.248)	**0.001**		
Pathologic metastasis status (M0/M1)	2.568 (1.599–4.122)	**<0.0001**	2.446 (1.511–3.959)	**0.0003**
Histological grade (W/D/M/D, P/D)*	1.530 (1.054–2.223)	**0.026**		
Tumor margin status (negative/positive)	1.295 (0.902–1.859)	0.161		
Nerve plexus invasion (absence/presence)*	1.545 (1.100–2.168)	**0.012**	1.459 (1.030–2.065)	**0.033**
Lymphatic invasion (0, 1/2, 3)*	1.945 (1.341–2.819)	**0.0005**		
Venous invasion (0, 1/2, 3)*	2.152 (1.530–3.027)	**<0.0001**	1.991 (1.408–2.815)	**<0.0001**
Intrapancreatic neural invasion (0, 1/2, 3)*	1.649 (1.190–2.287)	**0.003**		
Expression of ARG2 in PDC (absence/presence)	1.287 (0.859–1.928)	0.221		
Expression of ARG2 in stromal cells (absence/presence)	1.735 (1.262–2.384)	**0.0006**	1.715 (1.244–2.364)	**0.001**

W/D, well differentiated tubular adenocarcinoma and papillary carcinoma; M/D, moderately differentiated.

tubular adenocarcinoma; P/D, poorly differentaited adenocarcinoma.

* Classified according to the classification of pancreatic carcinoma of Japan Pancreas Society.

### Correlation between the Presence of ARG2-expressing Stromal Cells and Other Clinicopathological Variables

Table 3 lists the clinicopathological features of patients with PDC. When these features were analyzed for correlations, the presence of ARG2-expressing stromal cells was found to be more likely in cases with poorer tumor differentiation in terms of histological grade, presence of necrosis, and presence of stromal cells expressing CAIX and SLC2A1 (alternatively known as glucose transporter type 1, GLUT1), which are markers of hypoxia. In addition, the presence of ARG2-expressing stromal cells was closely correlated with higher tumor-infiltrating CD68^+^ macrophages and CD66b^+^ neutrophils, and lower tumor-infiltrating CD4^+^ T cells and CD8^+^ T cells. No significant correlation was found between the presence of ARG2-expressing cancer cells and any of these clinicopathological parameters.

**Table 3:** Correlation of expression of ARG2 in stromal cells with clinicopathological characteristics.

		Expression of ARG2							Expression of ARG2			
Characteristics	No. ofpatients	absence	presence	*P*	Characteristics	No. ofpatients			absence		presence	*P*
Age, years					Lymphatic invasion*							
<60	71	46	25		0, 1	63			38		25	
≥60	143	79	64	0.189	2, 3	151			87		64	0.762
Sex					Venous invasion*							
Male	130	70	60		0, 1	83			53		30	
Female	84	55	29	0.118	2, 3	131			72		59	0.205
Size (mm)					Intrapancreaticneural invasion*							
<30	50	32	18		0, 1	94			61		33	
≥30	164	93	71	0.414	2, 3	120			64		56	0.096
Pathologic tumor status					Histologicalnecrosis							
T1	2	2	0		Absence	77			61		16	
T2	2	0	2		Presence	137			64		73	**<0.0001**
T3	210	123	87		Expression of CAIX in stromal cells							
T4	0	0	0	0.120**	Absence		167		121		46	
Pathologic node status					Presence		47		4		43	**<0.0001**
N0	42	24	18		Expression of CAIX in cancer cells							
N1	172	101	71	0.863	Absence		111		68		43	
					Presence		103		57		46	0.407
M0	193	115	78		Expression of SLC2A1 (Glut-1) in stromal cells							
M1	21	10	11	0.353	Absence		97	89		8		
Stage					Presence		117	36		81		**<0.0001**
IA	1	1	0		Expression of SLC2A1 (Glut-1) in cancer cells							
IB	1	0	1		Absence		85	58		27		
IIA	40	23	17		Presence		129	67		62		**0.023**
IIB	151	91	60		Tumor-infiltrating CD68^+^ macrophages***							
III	0	0	0		lower		105	72		33		
IV	21	10	11	0.501**	higher		106	51		54		**0.005**
Tumor histological grade*					Tumor-infiltrating CD66b^+^ neutrophils***							
W/D	55	41	14		lower		105	76		29		
M/D	110	65	45		higher		106	48		58		**<0.0001**
P/D	49	19	30	**0.001** **	Tumor-infiltrating CD4^+^ T cells***							
Tumor margin status					lower		105	51		54		
Negative	159	94	65		higher		106	73		33		**0.003**
Positive	55	31	24	0.753	Tumor-infiltrating CD8^+^ T cells***							
Nerve plexusl invasion*					lower		105	54		51		
Absence	79	43	36		higher		106	70		36		**0.036**
Presence	135	82	53	0.391	Total		214	125		89		

W/D, well differentiated tubular adenocarcinoma and papillary carcinoma; M/D, moderately differentiated.

tubular adenocarcinoma; P/D, poorly differentaited adenocarcinoma.

* Classified according to the classification of pancreatic carcinoma of Japan Pancreas Society.

** Comparisons of qualitative variables are performed using the χ^2^ test and otherwise Fisher’s exact test.

*** Total number of patients are 211 and otherwise 214.

### Majority of ARG2-expressing Stromal Cells are Cancer-associated Fibroblasts (CAFs) in a Hypoxic State

**Figure 3:**
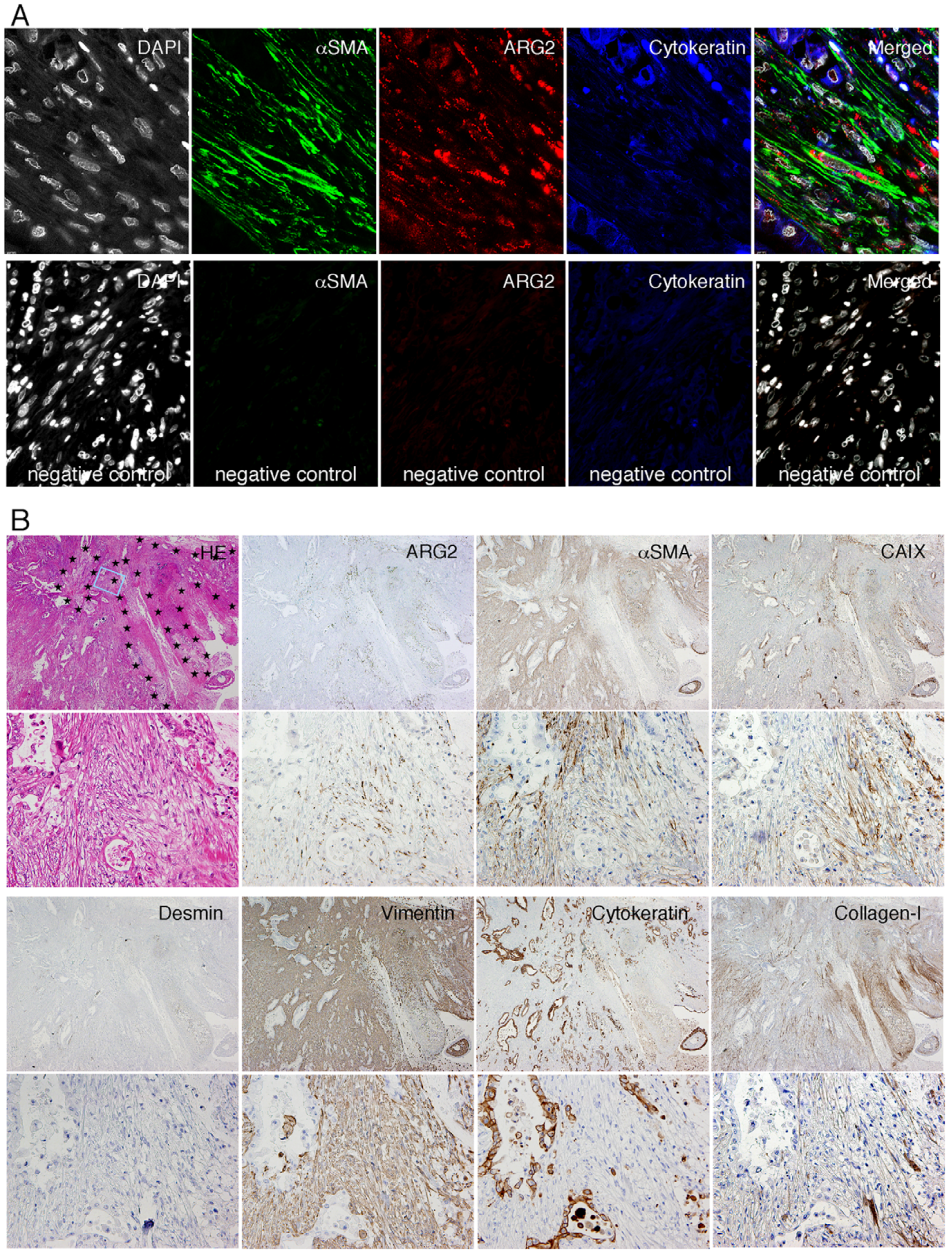
ARG2 was expressed mostly in CAFs within and around necrotic areas in PDC tissue. (A) Triple immunofluorescence shows that most of the dot-like staining of ARG2 (red) is present in α-SMA-positive fibroblasts (green). Cancer cells are positive for cytokeratins (blue). Nuclei are stained by DAPI (white). (B) Histology and immunohistochemistry of PDC tissue in low- (upper-photo of each pair of photos) and high-power view (lower-photo of each pair of photos). HE staining and immunohistochemistry for several antigens in serial tissue sections. Necrotic areas are surrounded by star marks in the low-power HE photo and the rectangle (light blue) corresponds to the area of the high-power view.

To determine what kind of stromal cells expressed ARG2, we performed double immunohistochemistry and triple immunofluorescence. ARG2-expressing spindle cells were often positive for α-SMA, vimentin, and collagen type I, rarely positive for CD31 and CD68 (data not shown), and negative for desmin, cytokeratins, and D2-40 (Figure 3), indicating that the majority of ARG2-expressing stromal cells were CAFs and a minority were endothelial cells or macrophages. However, most of the CAFs in PDC tissue did not express ARG2, except for those present within and around necrotic and myxoid degenerative areas. Surprisingly, ARG2 was not apparently expressed in the stromal or epithelial cells surrounding necrotic tissue or myxoid degenerative areas in intraductal papillary-mucinous neoplasms of the pancreas and necrotic tissue in peptic ulcers (data not shown).

**Figure 4:**
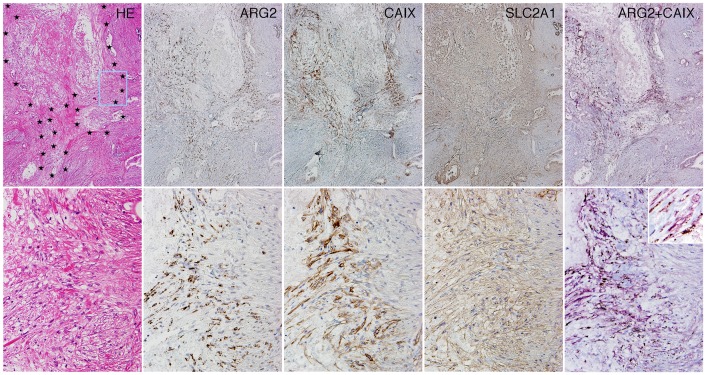
ARG2 was expressed mostly in CAFs under hypoxic conditions. Histology of PDC tissue in low- (upper columns) and high-power view (lower columns). HE staining and immunohistochemistry for ARG2, CAIX, and SLC2A1 in serial tissue sections. Necrotic areas are surrounded by star marks in the upper HE photo and the rectangle (light blue) corresponds to the area of the lower column. Double immunostaining (the right-most columns) reveals that most of the granular ARG2 staining (brown) is present in spindle-shaped cells stained for CAIX (purple). Inset is a very high-power view.

Since ARG2-expressing CAFs were present within and around necrotic areas, we next examined the relationship between ARG2 expression and hypoxia in cancer tissue using double immunostaining. Expression of CAIX, SLC2A1, or HIF-1α was observed in CAFs around areas of necrosis. ARG2 expression was found in these CAIX-, SLC2A1-, or HIF-1α-expressing CAFs themselves, or in CAFs located next to CAIX-, SLC2A1-, or HIF-1α-expressing cells (Figure 4). ARG2 expression in cancer cells was not restricted to areas around necrosis or near CAIX-, SLC2A1-, or HIF-1α-expressing cells (data not shown). These findings suggested that expression of ARG2 was induced in the CAFs under hypoxic conditions, and also that there was no apparent correlation between hypoxia and the expression of ARG2 in cancer cells.

### Hypoxia Induces Expression of ARG2 in CAFs Extracted from PDC Tissues

**Figure 5:**
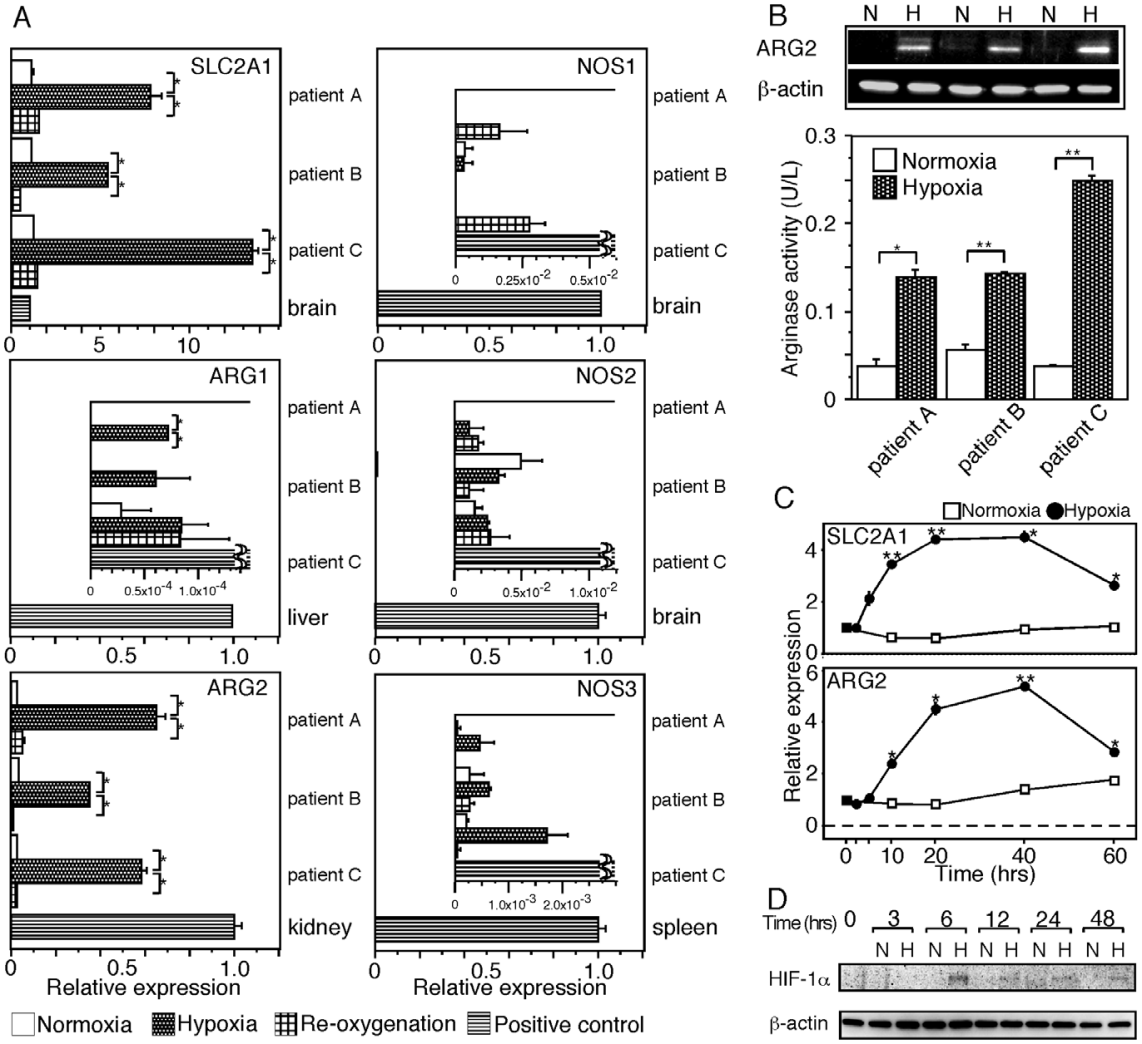
Hypoxia induces expression of *ARG2* in CAFs extracted from PDC tissue. (A) Relative gene expression is measured by real-time RT-PCR in fibroblasts extracted from PDC tissue after 48 hrs of exposure to hypoxia (Hypoxia) or culture under normoxic control conditions (Normoxia). Expression of the genes in the fibroblasts after 48 hrs of culture under hypoxic conditions followed by 48 hrs of culture under normoxic conditions (Re-oxygenation) was also analyzed. Each data column represents the mean relative expression standardized with 18SrRNA ± SE for triplicate determinations. The *SLC2A1* (alternatively known as glucose transporter type 1 or GLUT1) gene is hypoxia-inducible. Significance value (Student’s *t* test) of *P*<0.01 (*). Some genes are expressed in CAFs at an extremely lower level than in a normal tissue that is the major tissue of expressing the gene. In such cases, comparison of gene expression is shown in the insets with 1/50 to 1/10000 scales. (B) Western blot analysis (upper column) reveals that ARG2 protein expression is induced in CAFs used in (A) upon exposure to hypoxia. N and H indicate the cells cultured under normoxic and hypoxic conditions, respectively. ARG2 protein induced in CAFs has arginase activity (lower column). One unit of arginase converts 1 µmol of L-arginine to ornithine and urea per minute at pH 9.5 and 37°C. Each data column represents the mean activity ± SE for triplicate determinations. Significance value (Student’s *t* test) of *P*<0.05 (*) and *P*<0.01 (**). (C) Expression of genes in CAFs from PDC tissue during cultivation under hypoxic conditions detected by real-time RT-PCR. Data represent one of three independent experiments. Significance value (Student’s *t* test) of *P*<0.01 (*) and *P*<0.001 (**). (D) Western blot analysis of HIF-1α protein. Accumulation of HIF-1α protein is observed in CAFs after 6 hrs of exposure to hypoxic conditions. N and H indicate cells cultured under normoxic and hypoxic conditions, respectively.

To confirm if expression of the *ARG2* gene is induced by hypoxia in CAFs, we carried out *in vitro* experiments using CAFs extracted from PDC tissues. Induction of *ARG2* gene expression by hypoxic stress was found in CAFs at both the transcription and protein levels, and the induced ARG2 had arginase activity (Figures 5A–C). Expression of ARG2 continued under hypoxic conditions, and the induction of ARG2 by hypoxic stress was reversible. Re-oxygenation decreased the up-regulated expression of the *ARG2* gene to a level comparable to that in CAFs cultured under normoxic conditions (Figure 5A). Hypoxic stress induced accumulation of HIF-1α in CAFs (Figure 5D). According to the gene database, the 5′ flanking region of the first exon of the ARG2 gene has potential binding sites for HIF-1 (5′-RCGTG-3′) [13]: ACGTG at -425 to -421 and GCGTG at -234 to -230. These findings suggest that expression of ARG2 is induced via the HIF-1 signaling pathway.

Expression of the *ARG1*, *NOS1, NOS2*, and *NOS3* genes was not significantly induced by hypoxia (Figures 5A). Expression of the *ARG1* and *NOS3* genes seemed to be induced by hypoxia, although their levels of expression were very low, almost 1/1000 to 1/10000 of those in a normal tissue, which is one of the major tissues that express the gene. Since all of these genes encode enzyme molecules, it is unlikely that such small amounts of induced transcripts would be biologically important. Rather, it is speculated that small amounts of contaminating cells such as endothelial cells might have responded to the hypoxic conditions. Peroxynitrite is a reactive nitrogen intermediate produced when both NOS and ARG are present. Since these cells did not express nitrotyrosine examined by immunohistochemistry (data not shown) that is generated by the nitration of tyrosine residues by peroxynitrite, *NOS2* expression was not induced in CAFs within and around necrotic areas in PDC tissue. These results suggest that *NOS2* is not induced in *ARG2*-expressing CAFs.

### ARG2-expressing CAFs Potentially Affect the Immune Reaction

**Figure 6:**
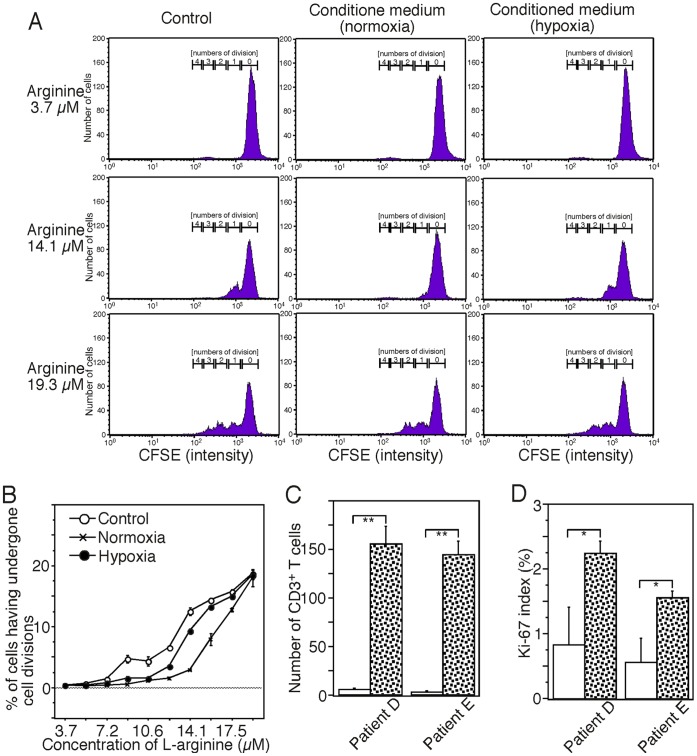
ARG2-expressing CAFs potentially affect the immune reaction. (A) T cell proliferation assay. CFSE-labeled CD3^+^ T cells were stimulated with anti-CD3/CD28 antibody-conjugated beads in fresh medium supplemented with the indicating L-arginine (control) or in conditioned medium after culture of CAFs under normoxic conditions (normoxia) or hypoxic conditions (hypoxia) in the medium with the indicating L-arginine (including 2.0 µM arginine of serum contents) for four days, and their proliferation profiles were analyzed by flow cytometry. Non-proliferated cells showed the highest fluorescence intensity and the intensity of the labeled T cells was halved with every cell division. The numbers represent the ratio of numbers of cells having undergone multiple divisions relative to the original number of cells. Data represent one of six independent experiments. (B) Percentages of cells having undergone cell divisions. Data represent one of six independent experiments. (C, D) Comparison of absolute number of tumor-infiltrating CD3^+^ T cells (C) with their proliferating index (the proportion of Ki-67-positive proliferating cells among the CD3^+^ T cells) (D) between the area around ARG2-expressing CAFs (white) and the area within the tumor except for necrotic tissue (speckled). We counted tumor-infiltrating CD3^+^ T cells and their Ki-67 positivity in each twenty different high-power fields per area categories using two PDC cases. Each data column represents the mean± SE for triplicate determinations. Significance value (Student’s *t* test) of *P*<0.05 (*) and *P*<0.001 (**).

The normal physiological concentration of L-arginine in serum is around 100 µM (50–150 µM). We determined that 12–15 µM L-arginine would be required for T cell proliferation induced by T cell receptor stimulation under the experimental conditions we employed (Figures 6A and 6B). Next, we tried to examine if ARG2 induced by exposure to hypoxia affects the proliferation of T cells *in vitro*. In contrary to the expectation, the conditioned medium that had been used for culturing CAFs under hypoxic conditions did not suppress T cell proliferation significantly in comparison to medium that had been used for culturing CAFs under normoxic conditions (Figure 6A and B). The discrepancy of the findings to our results of the induction of ARG2 protein with a certain enzymatic activity in CAFs by hypoxic exposure might be caused by the secretion of undetermined molecules that can support T cell proliferation from the CAFs.

In order to determine whether CD3^+^ T cells are proliferating around ARG2-expressing CAFs in PDC tissue, we performed double immunohistochemistry for CD3 and Ki-67 and compared tumor-infiltrating CD3^+^ T cells with their proliferating index in the area around ARG2-expressing CAFs to the area within the tumor except for necrotic tissue. It was surprised that there were few CD3^+^ T cells around ARG2-expressing CAFs. Both the absolute number of tumor-infiltrating CD3^+^ T cells (Figure 6C) and the proportion of Ki-67-positive proliferating cells among the CD3^+^ T cells (Figure 6D) observed in the area around ARG2-expressing CAFs were significantly lower than those observed in the other area. These findings suggest that the adaptive immune response is suppressed in areas around ARG2-expressing CAFs.

**Figure 7:**
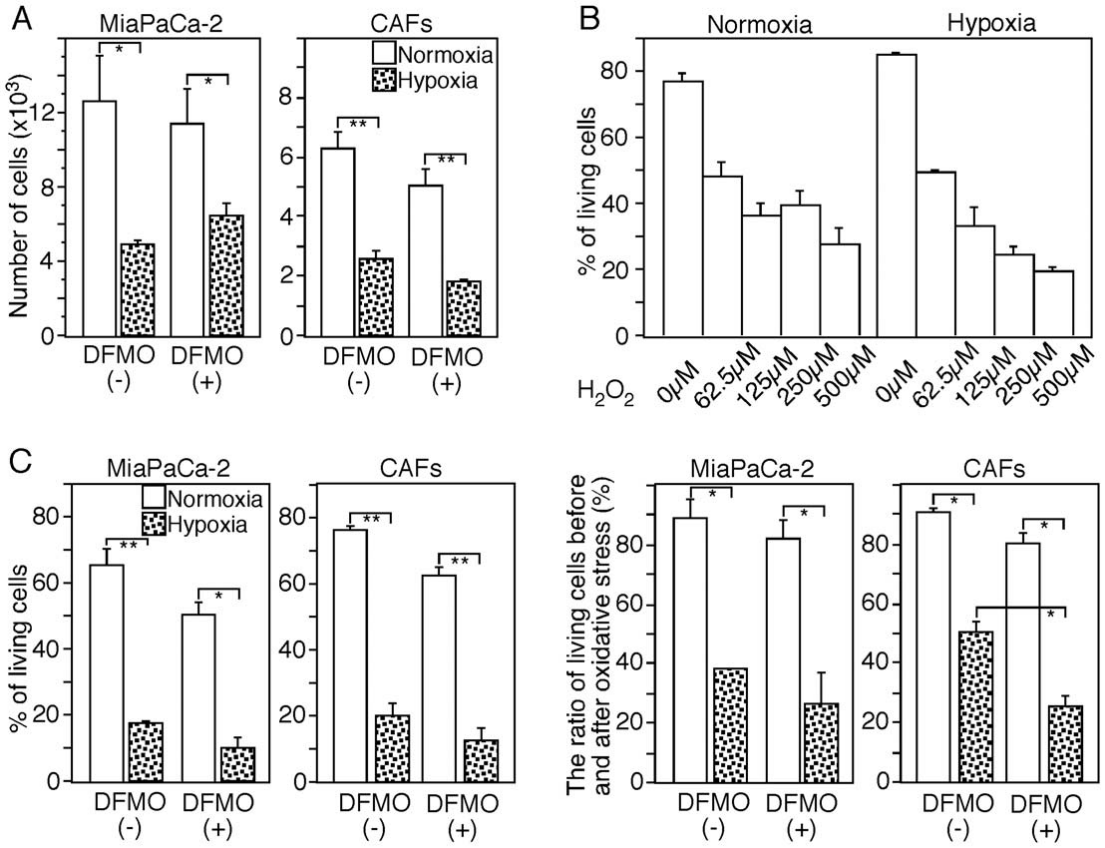
Pancreatic cancer cells and ARG2-expressing CAFs. (A) ARG2-expressing CAFs do not support proliferation of pancreatic cancer cells. CAFs extracted from PDC tissues and MiaPaCa-2 cells were co-cultured in medium with or without 2 mM DFMO under normoxic or hypoxic conditions for 48 hrs and the numbers of living cells were calculated the basis of data obtained by flow cytometry. The absolute number of MiaPaCa-2 cells cultured under hypoxic conditions decreased significantly in comparison with normoxic conditions, although this effect was not significantly affected by the presence of DFMO in the culture medium. Data represent one of three independent experiments. Significance value (Student’s *t* test) of *P*<0.05 (*) and *P*<0.01 (**). (B) Oxidative stress-induced apoptosis was induced in MiaPaCa-2 cells by exposure to various concentrations (0–500 µM) of H_2_O_2_ for 7 hrs. The dead cells and living cells were detected by flow cytometry after staining with Annexin V and PI. (C) ARG2-expressing CAFs did not protect pancreatic cancer cells from oxidative-induced apoptosis. After 48 hrs of co-culture of CAFs extracted from PDC tissues and MiaPaCa-2 cells in medium with or without 2 mM DFMO under normoxic or hypoxic conditions, all the cells were cultured for another 4 hrs under oxidative stress (50 µM H_2_O_2_) using the same conditions as before. The percentages of living cells were measured by flow cytometry (left column). In order to evaluate the effect of oxidative stress, the percentages of living cells after exposure to oxidative stress were divided by the percentages of living cells cultured under the same conditions before oxidative stress (right column). The ratio of living cells before and after oxidative stress decreased significantly in both MiaPaCa-2 cells and CAFs cultured under hypoxic conditions. Blocking the synthesis of polyamines with DFMO increased significantly the degree of oxidative stress-induced apoptosis in the CAFs. Data represent one of three independent experiments. Significance value (Student’s *t* test) of *P*<0.05 (*) and *P*<0.01 (**).

The direct effect of ARG2 induced by exposure to hypoxia in CAFs against cancer cells was investigated using an *in vitro* system in which CAFs extracted from PDC tissues were co-cultured with pancreatic cancer MiaPaCa-2 cells. The proliferation of cancer cells was not significantly affected by ARG2 induced by hypoxia (Figure 7A), and oxidative stress-induced apoptosis of cancer cells but CAFs themselves was not prevented by polyamine produced by ARG2 in response to hypoxia (Figure 7C).

## Discussion

ARG plays key roles in regulating most aspects of arginine metabolism in health and disease, as arginine is a precursor for several molecules with basically important biological functions, such as urea, nitric oxide, polyamines, proline, creatine, glutamate, and agmatine. [2], [4] Though ARG2 is reportedly involved in tumor immune escape mechanisms, [5] its roles in human cancer remain to be fully elucidated. Here we showed that the majority of PDCs did not express ARG2 and that the presence of ARG2-expressing PDCs was not significantly correlated with patient outcome. Instead, the presence of ARG2-expressing CAFs was found to be an independent predictor of poorer OS (HR  = 1.582, *P*  = 0.007) and DFS (HR  = 1.715, *P*  = 0.001) in PDC patients. The presence of ARG2 CAFs in PDC tissue was closely correlated with higher tumor grade, the presence of histological necrosis, the presence of CAIX-expressing stromal cells, and the presence of SLC2A1-expressing stromal cells in PDC tissue. ARG2-expressing CAFs were localized within and around necrotic areas in PDC tissue and co-expressed CAIX, SLC2A1, and HIF-1α. Furthermore, *in vitro* experiments revealed that cultured fibroblasts extracted from PDC tissue expressed the ARG2 after exposure to hypoxia. These results indicate that cancer cell-mediated immune suppression through ARG2 expression is not a general event and that the presence of ARG2-expressing CAFs is an independent prognostic factor for PDC patients, and also reflects tissue hypoxia.

Reduced oxygen tension has been reported to affect ARG activity in several tissues and cell types including lung, brain, endothelial cells, and macrophages, [14], [15], [16] although the effects on ARGs differ among tissues, cells, and species. In humans, it has been shown that hypoxia induces the expression of ARG2 in pulmonary artery smooth muscle cells (PASMC) [17] and lung microvascular endothelial cells. [18], [19] To our knowledge, the effects of hypoxia on ARG expression in cancer tissues or fibroblasts have not been demonstrated previously. Our study together with the study that hypoxia induced expression of ARG2 but not ARG1 in PASMCs suggests that ARG2 is the hypoxia-inducible isoform. [17] It is consistent with our findings that accumulation of HIF-1α was found in CAFs under hypoxic conditions and also that the 5' flanking region of the first exon of the ARG2 gene has potential binding sites for HIF-1.

Ornithine generated by ARG can be further metabolized to polyamines and proline, which are critical for cell proliferation, differentiation and tissue repair, thus potentially preventing cell death. [20], [21], [22] Therefore, induction of ARG2 might be a defensive response of CAFs in PDC tissue under severe hypoxic stress potentially leading to cell death. Indeed, our results showed that ARG2-expressing CAFs ameliorated oxidative stress-induced apoptosis of CAFs themselves (Figure 7C). This is consistent with that fact that these CAFs showed a high level of ARG and a low level of NOS, indicating extensive generation of polyamines and proline in them. These CAFs can also induce fibrosis, since proline is an essential component of collagen. As it has been observed that necrotic tissue is often remodeled to fibrous tissue as a form of healing response, [23] it is suggested that ARG2 induction is involved in fibrosis in hypoxic areas to facilitate post-necrotic fibrosis. Although it is still unclear if ARG2-expressing CAFs promote tumor progression, no significant effects on the proliferation of cancer cells and the protection of oxidative stress-induced apoptosis of cancer cells were found in our *in vitro* experiments (Figures 7A and 7C).

Most of the PDC cells did not express ARG2. Lung cancer expresses ARG2, although immune suppression mediated by ARG2-expressing cancer cells has not been observed. [6] Thus, it is suggested that cancer cell-mediated immune suppression by ARG2 (and NOS2) in prostate cancer [5] is probably tissue-dependent, and not a general event in the cancer microenvironment. Our investigation of tumor-infiltrating immune cells in PDCs showed that the presence of ARG2-expressing CAFs was significantly correlated with higher infiltration of CD68^+^ macrophages (*P*  = 0.05) and CD66b^+^ neutrophils (*P*<0.0001), and lower infiltration of CD4^+^ T cells (*P*  = 0.003) and CD8^+^ T cells (*P*  = 0.036). In addition, tumor-infiltrating CD3^+^ T cells around ARG2-expressing CAFs were few and showed less proliferation (Figure 6). These findings suggest that the presence of ARG2-expressing CAFs is closely related to the immunosuppressive microenvironment. This situation can be explained in terms of tissue hypoxia, [24] although the extent to which local expression of ARG2 in CAFs contributes to this immunosuppression is still unclear. A hypoxic environment attracts infiltrating innate immune cells, which negatively regulate adaptive immunity, [24] and hypoxia also promotes the microenvironment implicated in regulatory T cell development and function, [24] resulting in a strong impairment of T cell function. Immunosuppression induced by hypoxia and that mediated by ARG partly overlap. Hypoxic murine but not human macrophages express high levels of both ARG1 and NOS2 that lead to T cell suppression, where expression of both enzymes causes peroxynitrite generation, loss of CD3ζ chain expression, and T cell suppression and apoptosis. [25], [26] ARG1 acts mainly through depletion of arginine, which impairs T cell signal transduction and function. [27] Further studies are warranted to clarify whether and how ARG2 functions in immune editing in human cancer tissues.

Besides exerting host immunosuppressive effects, hypoxia can lead to the development of aggressive cancer phenotypes such as cell immortalization, autocrine growth/survival, angiogenesis, invasion/metastasis, and resistance to chemotherapy, through a mechanism mediated mainly by HIF-1α. [28], [29], [30] In the present study, we demonstrated that the presence of hypoxic foci containing ARG2-expressing CAFs was an independent predictor of poor survival in PDC patients, being consistent with our previous study in which the presence of necrosis was shown to be an independent predictor of poor outcome for such patients. [31] These findings and the therapeutic implications of hypoxia make it a high-priority target for cancer therapy. Recently, HIF-1-targeting therapy and anti-angiogenesis therapy have been reported to yield promising anti-cancer effects. [29], [30], [32] It is suggested that evaluation of ARG2-expressing CAFs would be useful not only for decision-making about postoperative clinical management, but also for stratifying patients for clinical trials aimed at evaluating HIF-1 targeting or anti-angiogenesis therapies.

## Materials and Methods

### Patients and Samples

This study was approved by the Institutional Review Board of the National Cancer Center, Japan (#17–77). Informed consent was obtained from all participants involved in this study (the consent was written) and all clinical investigation was conducted according to the principles expressed in the Declaration of Helsinki. Clinical and pathological data were obtained through a detailed retrospective review of the medical records of all 214 patients with ductal carcinoma of the pancreas who had undergone initial surgical resection between 1990 and 2005 at the National Cancer Center Hospital. None of the patients had received any prior therapy, and all had received standard therapy appropriate for their clinical stages. The operative procedures included 141 pancreatoduodenectomies or pylorus-preserving pancreatoduodenectomies, 56 distal pancreatectomies, and 7 total pancreatectomies. Secondary tumors and post-neoadjuvant cases were excluded. All patients had complete medical records, and had been followed in the tumor registries for survival and outcome. The clinicopathological characteristics of the patients are summarized in Table 3. One hundred thirty patients were male and 84 were female, with a mean age of 63.6 years (range, 27–87 years). Every patient was followed up in the outpatient clinic every 1–3 months during the first postoperative year, and every 6–12 months thereafter. No patient dropped out during follow-up. The median follow-up period after surgery was 18.8 (2.6–201) months for all patients. Forty-nine patients (20.4%) were alive at the census date (June 2009); 143 (66.8%) died of pancreatic cancer, and 22 (10.3%) died of other causes. Post-resection adjuvant therapy information was available for 208 patients, of whom 8 received chemotherapy and radiotherapy, 84 chemotherapy alone, and 2 radiotherapy alone; 113 did not receive any additional therapy.

### Pathological Examination

All of the ductal carcinomas were pathologically reexamined and classified according to the World Health Organization (WHO) classification, [9] the International Union against Cancer (UICC) tumor-node-metastasis (TNM) classification, [33] and the Classification of Pancreatic Carcinoma of the Japan Pancreas Society. [34] Surgically resected specimens were fixed in 10% formalin and cut into serial 5-mm-thick slices, horizontally in the pancreas head, and sagittally in the pancreas body and tail. All the sections were stained with hematoxylin and eosin (HE) for pathological examination.

### Immunohistochemistry

Immunohistochemistry was performed on formalin-fixed, paraffin-embedded tissue sections using the avidin-biotin complex method as described previously. [35] We used 4-µm-thick sections of representative blocks with antibodies against the following: ARG1 (poly H-52; 1∶200), ARG2 (poly H-64; 1∶200), nitrotyrosine (HM11; 1∶100), and CD3 (PS1; 1∶200) from Santa Cruz Biotechnology (Santa Cruz, CA), nitrotyrosine (poly; 1∶1000) from Millipore (Billerica, MA), CD31 (JC/70A; 1∶50), CD34 (QBEnd 10; 1∶100), CD68 (KP1; 1∶100), D2-40 (1∶100), cytokeratins (AE1/AE3; 1∶200), Ki-67 (MIB-1; 1∶100), α-smooth muscle actin (α-SMA, 1A4; 1∶50), desmin (D33; 1∶100), and vimentin (V9; 1∶500) from DAKO (Glostrup, Denmark), CAIX (M75, 1∶200), [36], [37] HIF-1α(54; 1∶500) from BD Transduction Laboratories (Franklin Lakes, NJ), CD4 (4B12; 1∶100) and CD8 (4B11; 1;200) from Leica Microsystems (Newcastle Upon Type, UK), CD66b (G10F5; 1∶200) from BioLegend (San Diego, CA), and mitochondria (MTC02; 1∶200) and collagen type I (COL-1; 1∶200) from Abcam (Cambridge, UK). For antigen retrieval, tissue sections were autoclaved at 121°C for 10 min in the Target Retrieval Solution (DAKO) for nitrotyrosine or in the buffer (20 mM Tris/HCl 1 mM EDTA, pH 9.0) for the other antigens. For semiquantitative assessments of the immunohistochemical results for ARG2, CAIX, and SLC2A1, we selected 10 randomized fields per tumor at a magnification of ×100, and the number of positive cells in each field was counted. The mean number of positive cells in the top three fields was calculated. Two observers (YI and NH), having no access to the patient data, independently evaluated these positively stained cells, and the final value employed was the average of the two counts they made. When the mean number of positive cells was zero, 1 to 80, and more than 80, the staining grades assigned were zero (absence), one (lower expression), and 2 (higher expression), respectively.

### Quantitative Evaluation of Tumor-infiltrating Immune/inflammatory Cells

After immunohistochemistry, the microscopic images were imported as digital photo files using a NanoZoomer Digital Pathology (NDP) system (Hamamatsu Photonics, Japan), and we selected three areas at low magnification in which the immunolabeled cells had infiltrated into tumor most densely. The selected areas were those in which it had been confirmed at high magnification that invasive proliferation of cancer cells was present. We did not select areas into which infiltrated immune/inflammatory cells had been recruited as a result of secondary tumor effects, such as pancreatitis, necrosis, ulceration, or mucus flooding of the tissue. Using NDP View at a magnification of ×200, immunolabeled lymphocytes, neutrophils (except intravascular neutrophils), or macrophages were then counted by two independent investigators (YI and NH). These observers were blinded to each other and also not provided with any clinical information on the outcome of the patients. The average counts for each of the three areas made by the two observers were then compared, and when the difference between their counts was less than 20% of the maximum value, the average of the two was used as the final count; if the difference exceeded 20%, the observers discussed the reasons for the difference and performed recounts until the difference became less than 20%. For the survival and correlation analyses, patients were divided into two groups showing high and low cell infiltration, representing values higher and lower than the median for tumor-infiltrating immune/inflammatory cells.

### Triple Immunofluorescence

Triple-immunofluorescence staining was performed on formalin-fixed paraffin-embedded sections as described previously with some modification. [38] The sections were reacted sequentially with a mixture of three primary antibodies (ARG2, cytokeratins, and one of CAIX, α-SMA, or CD31), a mixture of secondary antibodies (AlexaFluor488-conjugated anti-rabbit IgG antibodies, AlexaFluor647-conjugated anti-mouse IgG2a antibody, and biotin-conjugated anti-mouse IgG1 antibody), and Alexa-Fluor555-conjugated streptavidin (Invitrogen, Carlsbad, CA). Immunostained tissue sections were analyzed with a confocal microscope (LSM5 Pascal; Carl Zeiss, Germany) and a fluorescence microscope (BZ-9000; Keyence, Osaka, Japan).

### Extraction of Fibroblasts from PDC Tissue

Small pancreatic tissue blocks (100–150 mg) were obtained during pancreas surgery from patients with PDC. The tissue blocks were cut (1–5 mm^3^) and seeded in 6-cm^2^ culture dishes in the presence of Dulbecco's modified Eagle medium supplemented with 10% fetal calf serum and antibiotics. Tissue blocks were cultured at 37°C in 5% CO_2_ under a humidified atmosphere. Eighteen hours after seeding, the culture medium was changed, and 24 hours later, the small tissue blocks were transferred to new culture plates. The fibroblasts grew out in high number and purity from the tissue blocks 1 to 3 days later. The small tissue blocks were removed after 2–3 weeks. After reaching confluence, the monolayers were trypsinized and passaged 1∶2. The purity of the cells was assessed on the basis of morphology (most cells were stellate, with long cytoplasmic extensions; some were also spindle-shaped) and immunophenotypes (vimentin was expressed in 99.9% of the cells from all cases, α-SMA in 73–93%, cytokeratins in 0.4–0.5%, CD68 in 0.2–3%, desmin in 0%, and CD31 in 0–1%). Cell populations between passages 3 and 6 were used for the study.

### Quantitative RT-PCR (TaqMan) Analyses

After culture of the fibroblasts extracted from PDC tissue for 48 hrs under hypoxic [<1% O_2_, anaerobic chamber and AneroPack for Cell Gas generating system (Mitsubishi Gas Chemical)] [39] or normoxic conditions, gene expression in the cells was analyzed by real-time RT-PCR as described previously. [40] Briefly, single-stranded cDNA was synthesized from total RNA extracted from the fibroblasts. Quantitative RT-PCR for target genes and non-target housekeeping control genes was performed with the ABI Prism 7500 Sequence Detection System (Applied Biosystems) using FastStart Universal Probe Master (ROX) and probes from the Universal Probe Library (Roche Diagnostics Corp., Indianapolis, IN). The sequences of the primers and the respective Universal Probe Library probes were: 18SrRNA (probe ID #48, 5′: primer gcaattattccccatgaacg, 3′ primer: gggacttaatcaacgcaagc), ARG1 (probe ID #20, 5′: primer aggtctgtgggaaaagcaag, 3′ primer: gcttccaattgccaaactgt), ARG2 (probe ID #64, 5′: primer tgtgtcacactgggaggaga, 3′ primer: aacacaaaggtctgggcagt), NOS1 (probe ID #39, 5′: primer ctgggagactgaggtggttc, 3′ primer: agtgcatcccgtttccaa), NOS2 (probe ID #19, 5′: primer gccacagaagagcctgagag, 3′ primer: tggtgaacttccacttgctg), NOS3 (probe ID #5, 5′: primer gaccctcaccgctacaacat, 3′ primer: ccgggtatccaggtccat), and SLC2A1 (probe ID #67, 5′: primer ggttgtgccatactcatgacc, 3′ primer: cagataggacatccagggtagc).

### Western Blot Analysis

CAFs were lysed in a lysis buffer [1% Triton X-100 (Sigma-Aldrich) and a cocktail of proteinase inhibitors (Roche Diagnostics Corporation) in phosphate-buffered saline, pH 7.4]. Equal amounts of protein samples were separated on 7% or 10% polyacrylamide gels and transferred to Immobilon-P membranes (Millipore). The membranes were immersed in a blocking solution (5% skim milk in TBS-T) for at least 1 hr at 4°C, and then incubated overnight at 4°C with antibodies. After being washed, the membranes were incubated for 30 min at room temperature with peroxidase-conjugated anti-mouse IgG (ab′)_2_ fragment (Amersham, Arlington Heights, IL). The antigen was detected with enhanced chemiluminescence Western blotting detection reagents (Amersham) according to the manufacturer’s instructions.

### Arginase Enzyme Activity

After cultivating under normoxic condition or hypoxic condition for 48 hrs, CAFs were lysed in a lysis buffer [0.4% Triton X-100, 150 mM NaCl, and a cocktail of proteinase inhibitors in 50 mM Tris/HCl, pH 7.6]. Arginase enzyme activities of their lysates were measured by QuantiChrom Arginase Assay Kit (BioAssay Systems, Hayward, CA) according to the manufacturer's instructions.

### T Cell Proliferation Assay

Human CD3^+^ T cells were purified from healthy volunteer peripheral blood by Ficoll density gradient centrifugation, followed by isolation with immunomagnetic beads (Miltenyi Biotec, Bergisch Gladbach, Germany). Purity was >95%, as confirmed by flow cytometry. 1×10^5^ of the T cells were labeled by 5-(and-6)-carboxyfluorescein diacetate succinimidyl ester (CFSE) (Invitrogen) according to the manufacturer's instructions, stimulated with anti-human CD3/CD28 antibody-conjugated beads (Invitrogen) in arginine-free medium [arginine/leucine/lysine-free RPMI-1640 (Sigma) supplemented with 2% human AB serum, 50 mg/l of L-leucine, 40 mg/l of L-lysine/HCl, and penicillin-streptomycin-glutamine (Invitrogen)] with or without L-arginine for four days, and their proliferation profiles were analyzed by flow cytometry. The conditioned medium after culture of 1×10^5^ CAFs in a 48-well dish under normoxic or hypoxic conditions in the arginine-free medium with the indicating L-arginine for four days were used instead of the fresh control medium.

### Cancer Cell Proliferation and Apoptosis Assay

Ten thousand CAFs were cultured in a 48-well dish for 48 hrs, then 2×10^4^ human pancreatic cancer MiaPaCa-2 cells (American Type Culture Collection, Manassas, VA) were seeded on the CAFs and co-culture was carried out for 48 hrs under normoxic or hypoxic conditions. The cells were usually cultured in RPMI-1640 with 10% fetal bovine serum and penicillin-streptomycin-glutamine (Invitrogen) at 37°C in a humidified incubator with 5% CO_2_. It is thought that the biological activity of ARG2 is exerted mainly through polyamines, which are metabolites of arginine produced by ARG2 and several other enzymes, including ornithine decarboxylase (ODC). In order to block the biological function of ARG2, we used an ODC inhibitor, alpha-difluoromethylornithine (DFMO) (Sigma). CAFs were precultured in fresh medium with 2 mM DFMO for two days and then co-cultured with MiaPaCa-2 cells in the same medium under normoxic and hypoxic conditions. In order to measure the numbers of both living and apoptotic cells, the cells were trypsinized, and reacted sequentially with a mixture of two primary antibodies [CD44v5 (VFF-8; 1∶200, Bender MedSystems, Vienna, Austria) and Emmprin (poly sc-9752; 1∶100, Santa Cruz Biotechnology)], a mixture of two secondary antibodies [biotin-conjugated anti-mouse IgG antibodies and biotin-conjugated anti-goat IgG antibodies (Vector, Burlingame, CA)] and AlexaFluor488-conjugated streptavidin (Invitrogen). After changing the buffer to Annexin V binding buffer (10 mM HEPES, pH 7.4, supplemented with 140 mM NaCl, 2 mM CaCl_2_, and 1% bovine serum albumin), the cells were reacted with AlexaFluor647-conjugated Annexin V (BioLegend) and propidium iodide (PI, Sigma). Flow cytometry analyses were carried out using a FACSCalibur (BD Biosciences, San Jose, CA) and the data were analyzed using CellQuestPro software (BD Biosciences). In our preliminary tests, MiaPaCa-2 expressed CD44v5 and Emmprin but not CEA (Ab-3; 1∶50, Lab Vision, Kalamazoo, MI), CA19-9 (C241∶5:1∶4; 1∶200, Leica Microsystems), MUC1 (Ma552; 1∶100, Leica Microsystems), epithelial membrane antigen (E29∶1:100, DAKO), and EpCAM (G8.8; 1∶200, Santa Cruz Biotechnology), and CAFs did not express CD44v5 and Emmprin. For the oxidative stress assay, cells were cultured in the normal medium with 50 µM of H_2_O_2_ for four hrs and we analyzed cell death and apoptosis of them by flowcytometer.

### Statistical Analysis

Comparisons of qualitative variables were performed using the χ^2^ test or Fisher’s exact test. One-way analysis of variance (ANOVA) was used to compare the means of three or more groups. The postoperative disease-free survival (DFS) and overall survival (OS) rates were calculated by the Kaplan-Meier method. Univariate analysis was performed for prognostic factors using the log-rank test. The factors found to be predictive by univariate analysis were subjected to multivariate analysis using the Cox proportional hazards model (backward elimination method). Differences at *P*<0.05 were considered statistically significant. Statistical analyses were performed with StatView-J 5.0 software package (Abacus Concepts, Berkeley, CA).

## References

[1] Hanahan D, Weinberg RA (2011) Hallmarks of cancer: the next generation. Cell 144: 646–674. 21376230 10.1016/j.cell.2011.02.013

[2] Munder M (2009) Arginase: an emerging key player in the mammalian immune system. Br J Pharmacol 158: 638–651. 19764983 10.1111/j.1476-5381.2009.00291.x PMC2765586

[3] Rodriguez PC, Zea AH, DeSalvo J, Culotta KS, Zabaleta J, et al (2003) L-arginine consumption by macrophages modulates the expression of CD3 zeta chain in T lymphocytes. J Immunol 171: 1232–1239. 12874210 10.4049/jimmunol.171.3.1232

[4] Morris SM Jr (2009) Recent advances in arginine metabolism: roles and regulation of the arginases. Br J Pharmacol 157: 922–930. 19508396 10.1111/j.1476-5381.2009.00278.x PMC2737650

[5] Bronte V, Kasic T, Gri G, Gallana K, Borsellino G, et al (2005) Boosting antitumor responses of T lymphocytes infiltrating human prostate cancers. J Exp Med 201: 1257–1268. 15824085 10.1084/jem.20042028 PMC2213151

[6] Rotondo R, Mastracci L, Piazza T, Barisione G, Fabbi M, et al (2008) Arginase 2 is expressed by human lung cancer, but it neither induces immune suppression, nor affects disease progression. Int J Cancer 123: 1108–1116. 18528866 10.1002/ijc.23437

[7] Siegel R, Ward E, Brawley O, Jemal A (2011) Cancer statistics, 2011: the impact of eliminating socioeconomic and racial disparities on premature cancer deaths. CA Cancer J Clin 61: 212–236. 21685461 10.3322/caac.20121

[8] Center for Cancer Control and Information Services, National Cancer Center J (2009) Cancer Statistics in Japan.

[9] Hruban RH, Boffetta P, Hiraoka N, Iacobuzio-Donahue C, Kato Y, et al.. (2010) Ductal adenocarcinoma of the pancreas. In: Bosman FT, Carneiro F, Hruban RH, Theise ND, editors. World Health Organization Classification of Tumours Pathology & Genetics Tumours of the Digestive System. 4th ed. Lyon: IARCPress. 281–291.

[10] Hruban RH, Pitman MB, Klimstra DS (2007) Ductal adenocarcinoma. In: Hruban RH, Pitman MB, Klimstra DS, editors. AFIP Atlas of Tumor Pathology Tumors of the pancreas. 4th ed. Washington, DC.: ARP Press. 111–164.

[11] Lim JE, Chien MW, Earle CC (2003) Prognostic factors following curative resection for pancreatic adenocarcinoma: a population-based, linked database analysis of 396 patients. Ann Surg 237: 74–85. 12496533 10.1097/00000658-200301000-00011 PMC1513971

[12] Philip PA, Mooney M, Jaffe D, Eckhardt G, Moore M, et al (2009) Consensus report of the national cancer institute clinical trials planning meeting on pancreas cancer treatment. J Clin Oncol 27: 5660–5669. 19858397 10.1200/JCO.2009.21.9022 PMC7587401

[13] Wenger RH, Stiehl DP, Camenisch G (2005) Integration of oxygen signaling at the consensus HRE. Sci STKE 2005: re12. 16234508 10.1126/stke.3062005re12

[14] Albina JE, Henry WL Jr, Mastrofrancesco B, Martin BA, Reichner JS (1995) Macrophage activation by culture in an anoxic environment. J Immunol 155: 4391–4396. 7594599

[15] Louis CA, Reichner JS, Henry WL Jr, Mastrofrancesco B, Gotoh T, et al (1998) Distinct arginase isoforms expressed in primary and transformed macrophages: regulation by oxygen tension. Am J Physiol 274: R775–782. 9530245 10.1152/ajpregu.1998.274.3.R775

[16] Clarkson AN, Liu H, Rahman R, Jackson DM, Appleton I, et al (2005) Clomethiazole: mechanisms underlying lasting neuroprotection following hypoxia-ischemia. FASEB J 19: 1036–1038. 15809357 10.1096/fj.04-3367fje

[17] Chen B, Calvert AE, Cui H, Nelin LD (2009) Hypoxia promotes human pulmonary artery smooth muscle cell proliferation through induction of arginase. Am J Physiol Lung Cell Mol Physiol 297: L1151–1159. 19801451 10.1152/ajplung.00183.2009

[18] Krotova K, Patel JM, Block ER, Zharikov S (2010) Hypoxic upregulation of arginase II in human lung endothelial cells. Am J Physiol Cell Physiol 299: C1541–1548. 20861464 10.1152/ajpcell.00068.2010 PMC3774096

[19] Toby IT, Chicoine LG, Cui H, Chen B, Nelin LD (2010) Hypoxia-induced proliferation of human pulmonary microvascular endothelial cells depends on epidermal growth factor receptor tyrosine kinase activation. Am J Physiol Lung Cell Mol Physiol 298: L600–606. 20139181 10.1152/ajplung.00122.2009 PMC2853344

[20] Gerner EW, Meyskens FL Jr (2004) Polyamines and cancer: old molecules, new understanding. Nat Rev Cancer 4: 781–792. 15510159 10.1038/nrc1454

[21] Wu G, Morris SM Jr (1998) Arginine metabolism: nitric oxide and beyond. Biochem J 336 (Pt 1): 1–17. 10.1042/bj3360001 PMC1219836 9806879

[22] Li H, Meininger CJ, Hawker JR Jr, Haynes TE, Kepka-Lenhart D, et al (2001) Regulatory role of arginase I and II in nitric oxide, polyamine, and proline syntheses in endothelial cells. Am J Physiol Endocrinol Metab 280: E75–82. 11120661 10.1152/ajpendo.2001.280.1.E75

[23] Hasebe T, Tsuda H, Hirohashi S, Shimosato Y, Iwai M, et al (1996) Fibrotic focus in invasive ductal carcinoma: an indicator of high tumor aggressiveness. Jpn J Cancer Res 87: 385–394. 8641970 10.1111/j.1349-7006.1996.tb00234.x PMC5921099

[24] Sica A, Melillo G, Varesio L (2011) Hypoxia: a double-edged sword of immunity. J Mol Med (Berl) 89: 657–665. 21336851 10.1007/s00109-011-0724-8

[25] Sica A, Bronte V (2007) Altered macrophage differentiation and immune dysfunction in tumor development. J Clin Invest 117: 1155–1166. 17476345 10.1172/JCI31422 PMC1857267

[26] Gabrilovich DI, Nagaraj S (2009) Myeloid-derived suppressor cells as regulators of the immune system. Nat Rev Immunol 9: 162–174. 19197294 10.1038/nri2506 PMC2828349

[27] Ochoa AC, Zea AH, Hernandez C, Rodriguez PC (2007) Arginase, prostaglandins, and myeloid-derived suppressor cells in renal cell carcinoma. Clinical Cancer Research 13: 721 s–726 s. 10.1158/1078-0432.CCR-06-2197 17255300

[28] Semenza GL (2006) Development of novel therapeutic strategies that target HIF-1. Expert Opin Ther Targets 10: 267–280. 16548775 10.1517/14728222.10.2.267

[29] Grothey A, Galanis E (2009) Targeting angiogenesis: progress with anti-VEGF treatment with large molecules. Nat Rev Clin Oncol 6: 507–518. 19636328 10.1038/nrclinonc.2009.110

[30] Semenza GL (2009) HIF-1 inhibitors for cancer therapy: from gene expression to drug discovery. Curr Pharm Des 15: 3839–3843. 19671047 10.2174/138161209789649402

[31] Hiraoka N, Ino Y, Sekine S, Tsuda H, Shimada K, et al (2010) Tumour necrosis is a postoperative prognostic marker for pancreatic cancer patients with a high interobserver reproducibility in histological evaluation. British Journal of Cancer 103: 1057–1065. 20736942 10.1038/sj.bjc.6605854 PMC2965866

[32] Sessa C, Guibal A, Del Conte G, Ruegg C (2008) Biomarkers of angiogenesis for the development of antiangiogenic therapies in oncology: tools or decorations? Nat Clin Pract Oncol 5: 378–391. 18560389 10.1038/ncponc1150

[33] Sobin LH, Gospodarowicz MK, Wittekind C (2009) UICC TNM Atlas. Hoboken, NJ: Wiley-Blackwell.

[34] Japan-Pancreas-Society (2003) Classification of Pancreatic Cancer. Tokyo, Japan: Kanehara.

[35] Hiraoka N, Onozato K, Kosuge T, Hirohashi S (2006) Prevalence of FOXP3+ regulatory T cells increases during the progression of pancreatic ductal adenocarcinoma and its premalignant lesions. Clinical Cancer Research 12: 5423–5434. 17000676 10.1158/1078-0432.CCR-06-0369

[36] Pastorekova S, Zavadova Z, Kostal M, Babusikova O, Zavada J (1992) A novel quasi-viral agent, MaTu, is a two-component system. Virology 187: 620–626. 1312272 10.1016/0042-6822(92)90464-z

[37] Zavada J, Zavadova Z, Pastorek J, Biesova Z, Jezek J, et al (2000) Human tumour-associated cell adhesion protein MN/CA IX: identification of M75 epitope and of the region mediating cell adhesion. British Journal of Cancer 82: 1808–1813. 10839295 10.1054/bjoc.2000.1111 PMC2363230

[38] Takahashi Y, Akishima-Fukasawa Y, Kobayashi N, Sano T, Kosuge T, et al (2007) Prognostic value of tumor architecture, tumor-associated vascular characteristics, and expression of angiogenic molecules in pancreatic endocrine tumors. Clinical Cancer Research 13: 187–196. 17200354 10.1158/1078-0432.CCR-06-1408

[39] Okami J, Simeone DM, Logsdon CD (2004) Silencing of the hypoxia-inducible cell death protein BNIP3 in pancreatic cancer. Cancer Res 64: 5338–5346. 15289340 10.1158/0008-5472.CAN-04-0089

[40] Hiraoka N, Yamazaki-Itoh R, Ino Y, Mizuguchi Y, Yamada T, et al (2011) CXCL17 and ICAM2 are associated with a potential anti-tumor immune response in early intraepithelial stages of human pancreatic carcinogenesis. Gastroenterology 140: 310–321. 20955708 10.1053/j.gastro.2010.10.009

